# Sensitivity to Acceleration in the Human Early Visual System

**DOI:** 10.3389/fpsyg.2017.00925

**Published:** 2017-06-06

**Authors:** Ryohei Nakayama, Isamu Motoyoshi

**Affiliations:** Department of Life Sciences, The University of TokyoTokyo, Japan

**Keywords:** visual acceleration, velocity modulation sensitivity, attentive tracking, bandpass mechanism, visual search

## Abstract

It is widely believed that the human visual system is insensitive to acceleration in moving stimuli. This notion is supported by evidence that detection sensitivity for velocity modulation in moving stimuli is a lowpass function of the velocity modulation's temporal frequency. However, the lowpass function might be a mixture of detection by attention-based tracking and low-level mechanisms sensitive to acceleration. To revisit the issue of acceleration perception in relation to attentive tracking, we measured detection sensitivities for velocity modulations at various temporal frequencies (0.25–8 Hz) by using drifting gratings within long or short spatial windows that make the tracking of grating easier or more difficult respectively. Results showed that modulation sensitivity is lowpass for gratings with long windows but bandpass for gratings with short windows (peak at ~1 Hz). Moreover, we found that lowpass sensitivity becomes bandpass when we removed observer attention by a concurrent letter identification task. An additional visual-search experiment showed that a target dot moving with a velocity modulation at relatively high temporal frequencies (~2–4 Hz) was most easily detected among dots moving at various constant velocities. These results support the notion that high sensitivity to sluggish velocity modulation is a product of attentively tracking of moving stimuli and that the visual system is directly sensitive to accelerations and/or decelerations at the preattentive level.

## Introduction

It is widely believed that the human visual system, while remarkably sensitive to image motion, is little or not sensitive to temporal changes in motion speed, i.e., acceleration and deceleration (Gottsdanker, 1956; Mckee and Nakayama, 1988; Watamaniuk and Duchon, 1992). This idea is notably supported by psychophysical findings that detection sensitivity for velocity modulations in moving stimuli is a lowpass function of the modulation's temporal frequency (Snowden et al., 1991; Werkhoven et al., 1992). There has been no evidence for a bandpass function that effectively demonstrates a sharp sensitivity for acceleration. Thus, human observers are more sensitive to sluggish changes than to rapid changes in speed. These findings have been interpreted as indication that the visual system has no hard-wired acceleration detectors to directly process the temporal rate of velocity modulation (c.f., Schlack et al., 2007, 2008) and that it detects relatively slow changes in velocity by cognitively comparing perceived speeds at different moments in time.

Alternatively, recent psychophysical and electrophysiological studies suggest that visual motion detectors have a biphasic temporal response to motion inputs; i.e., motion detectors exhibit an excitatory response followed by an inhibitory response to subsequent motion signals. This biphasic characteristic is demonstrated by findings that very brief adaptations produce motion aftereffects (Kanai and Verstraten, 2005; Pavan et al., 2009; Glasser et al., 2011) and that direction selectivity in MT cells is inverted during the inhibitory response (Glasser et al., 2011). The analysis of involuntary ocular movements also indicates a similar biphasic temporal response to apparent motion over the visual field (Ohnishi et al., 2016). Theoretically, such a biphasic response in early motion detectors (Campana et al., 2011; Pavan and Skujevskis, 2013; Oluk et al., 2016) is computationally equivalent to a temporal derivative operation on motion signals. This line of evidence suggests that the visual system is directly sensitive to acceleration at some level of processing.

If we assume a linear system, as we argue above, detection sensitivity for velocity modulation will be a bandpass function of velocity modulation frequency. Several previous examinations of velocity modulation sensitivity employed single objects like dots that move along a straight path (Werkhoven et al., 1992). Such a stimulus can be easily tracked by attention especially if its speed is relatively slow (Verstraten et al., 2000). It is therefore possible that attentive tracking of the moving stimulus improves detection of slow-rate velocity modulation and results in the lowpass velocity-modulation detection function.

To test for this possibility, the present study re-examined the detection sensitivity function of velocity modulations by using a drifting grating in which attentive tracking is easy if the window is spatially elongated along the direction of movement but hard if it is short (Experiment 1). Results showed that the modulation sensitivity function is lowpass for stimuli with long windows but bandpass for stimuli with short windows with peak modulation sensitivity near 1 Hz. Moreover, we found that, even for long-window stimuli, the lowpass sensitivity curve becomes bandpass if observer attention is removed by a concurrent letter identification task (Experiment 2). In a subsequent experiment, observers searched a target defined by velocity modulation against a background of distracters with constant velocities and found that search performance is bandpass with a peak around 2 Hz (Experiment 3). Collectively, these results support the conclusion that attentive tracking plays a crucial role in the detection of sluggish velocity modulations. Under circumstances in which attentive tracking is difficult, the visual system exhibits peak sensitivity to velocity modulations at around 1–2 Hz. We suggest that the visual system is directly sensitive to acceleration/deceleration at the preattentive level and that this sensitivity is potentially mediated by biphasic motion detectors.

## Experiment 1

In the first experiment, we measured detection sensitivities for several velocity modulations of a grating drifting within either a long or short spatial window. The long- window stimulus tends to facilitate the attentive tracking of the grating (Mueller et al., 2016) and the detection of slow-rate velocity modulation (c.f., Verstraten et al., 2000). Therefore, a lowpass sensitivity function is expected for gratings with long windows. In contrast, the short window tends to prevent observers from continuously tracking the drifting grating (Mueller et al., 2016). If the lowpass sensitivity function mentioned above (Snowden et al., 1991; Werkhoven et al., 1992) is entirely due to the visual system's insensitivity to acceleration, then sensitivity would be expected remain lowpass. However, if we assume a linear system as well as low-level mechanisms sensitive to acceleration, we would then expect sensitivity to be bandpass.

### Methods

#### Observers

Three naive participants and one of the authors (RN) participated in Experiment 1 and 2. Three of those participants and two others took part in Experiment 3. All participants had corrected-to-normal vision. All the experiments were conducted in accordance with the Declaration of Helsinki and were approved by the ethics committee of the University of Tokyo. All participants provided written informed consent.

#### Apparatus

Images were displayed on a gamma-corrected 24-inch LCD (BENQ XL2430T; 640 × 480 pixel) with a frame rate of 120 Hz. This monitor is a successor to the BENQ XL2410T that has been proven to have sufficient temporal precision to display fast-changing stimuli common in visual psychophysics (Lagroix et al., 2012). The LCD's pixel resolution was 2.1 min/pixel at a viewing distance of 100 cm and mean luminance was 62.5 cd/m^2^.

#### Stimuli

**Figure 1:**
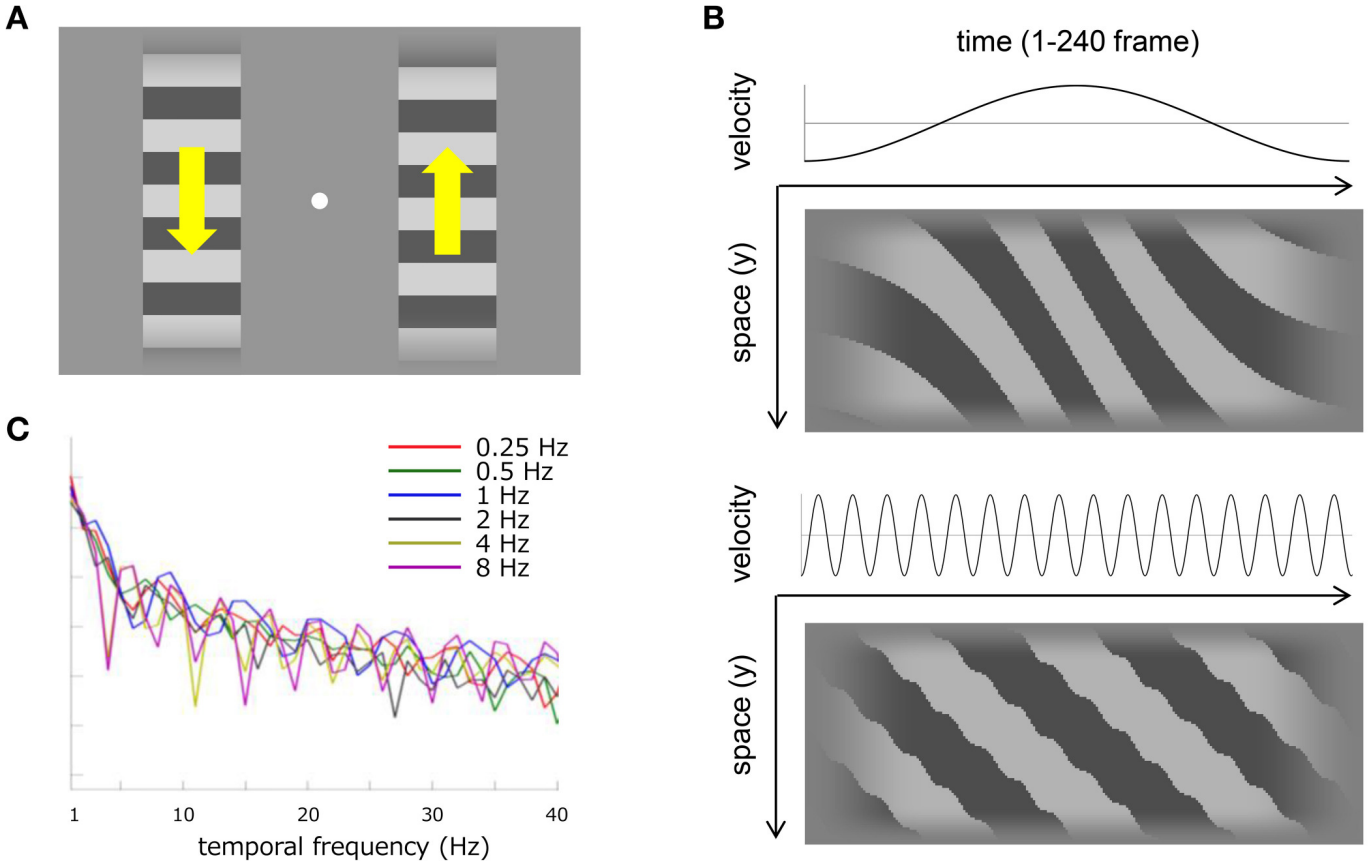
Visual stimuli and stimulus analyses. **(A)** Schematic of the stimulus display used in Experiment 1 and 2. Horizontal square-wave gratings were displayed on each side of fixation (white dot). The spatial window was either square or elongated along the drifting direction (as illustrated here). The gratings drifted vertically but always in opposite directions to each other as denoted by yellow arrows. One of the gratings drifted at a uniform velocity of 10.7 Hz and the other drifted with a sinusoidal modulation around 10.7 Hz. The temporal frequency of the velocity modulation varied from 0.25 to 8 Hz. **(B)** The top two panels illustrate 0.5 Hz velocity modulation and the bottom two panels illustrate 8 Hz velocity modulation. Both upper panels show a temporal change of velocity along sinusoidal profile with a mean velocity of 22.7 degree/s. Both lower panels show a temporal change of luminance in the vertical dimension of the horizontal grating within the short spatial window. One horizontal pixel corresponds to one frame in the stimulus presentation. **(C)** Temporal-frequency spectrum of the velocity modulation at a sampling frequency of 120 Hz. Each color corresponds to a different modulation frequency.

Visual stimuli consisted of a pair of horizontal square-wave gratings (0.5 cycle/degree), each of which was spatially defined by a window that was either square (H3.2 degree × V3.2 degree) or vertically elongated (H3.2 degree × V17.0 degree) as sketched in Figure 1A. The spatial windows were positioned 2.1° on each side of a fixation point (0.15° in diameter: 125.1 cd/m^2^) in the center of the screen. Gratings had a luminance contrast of 0.4 and horizontal edges were blurred vertically over a spatial extent of 0.5° by a cosine ramp that faded out smoothly into the mean luminance background. Gratings drifted at a temporal frequency of 10.7 Hz, and either one grating or the other underwent a sinusoidal velocity modulation at one of several temporal frequencies (0.25–8 Hz) as illustrated in velocity-time and space-time plots in Figure 1B. The temporal-frequency spectrum of the velocity modulation has several sharp troughs (especially for the highest velocity modulation) but generally fluctuates within a close range to each other modulation frequency (Figure 1C). Detection of the velocity modulation should not depend critically on artifacts from temporal aliasing attributable to the fixed frame rate (Figure 1B) or to sideband components resulting from frequency modulation (Figure 1C). The left- and right-side gratings always drifted in opposite directions, and drifting direction alternated between trials in order to minimize motion adaptation. Combinations of modulation frequencies and window sizes were interleaved in each experimental block. The spatial phase of the gratings and the temporal phase of the velocity modulation were randomized with the one exception that the initial phase angle for the 0.25 Hz frequency was randomly assigned to either 90° (deceleration) or 270° (acceleration) in order to display both of the fastest and slowest velocities in the half-cycle sinusoidal modulation that covered the stimulus duration of 2 s.

#### Procedure

The experiment was conducted in a dark room. On each trial, gratings were presented for 2 s and the presentation was temporally blurred by a cosine ramp within 0.4 s from the onset and offset. Observers were asked to gaze at fixation and indicate with the press of a button which of the two gratings underwent velocity modulation. Auditory feedback was given on correct and incorrect responses. The velocity modulation depth was determined for each trial according to an adaptive staircase method. After measurements, velocity modulation detection thresholds corresponding to 75% correct were estimated by means of a maximum-likelihood method.

### Results

**Figure 2:**
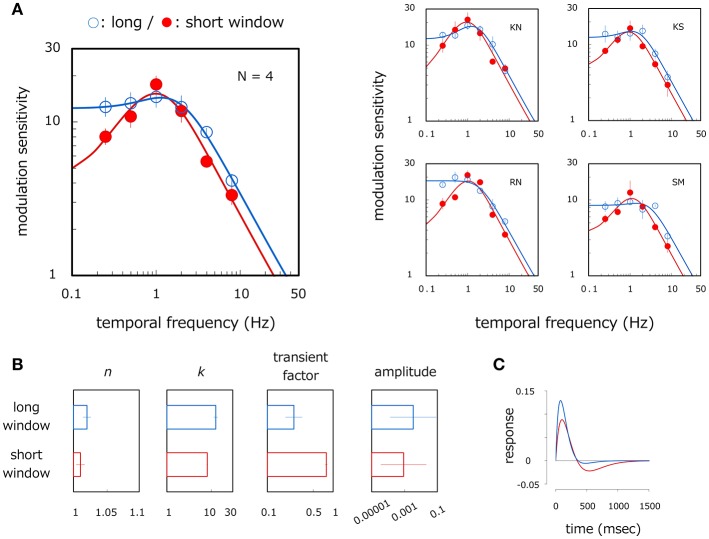
Results of Experiment 1. **(A)** Velocity modulation sensitivity for grating stimuli of different window sizes. Left panel shows results averaged across observers and right panels show those for each observer. Open and filled circles show modulation sensitivity as a function of the temporal frequency of velocity modulation for long- and short-window conditions respectively. Each curve represents the modulation transfer function (MTF) fitted to the plotted data. Error bars represent ±1 SE across observers or 400 bootstrap samples within observer. **(B)** Estimated parameters of the MTF for the long- and short-window conditions. Each panel represents *n, k*, transient factor, and amplitude. Error bars represent ±1 SE across observers. **(C)** Temporal impulse response function. Blue and red curves were estimated with the inverse Fourier transform of the MTF fitted to the mean data in the long- and short-window conditions respectively. The ordinate represents relative strength of system response where positive and negative values correspond to excitatory and negative response respectively.

The left panel of Figure 2A shows average velocity modulation sensitivity as a function of modulation temporal frequency. Open and filled circles show results for the long- and short-window conditions respectively. The smooth curve is the best-fitting conventional modulation transfer function (MTF)—described later in more detail—to the measured data. A two-way ANOVA reveals the main effect of the modulation frequency [ANOVA: *F*_(5, 15)_ = 134.264, *p* < 0.001] and the window size [ANOVA: *F*_(1, 3)_ = 29.447, *p* = 0.012], and the interaction between them [ANOVA: *F*_(5, 15)_ = 4.350, *p* = 0.012]. The modulation sensitivity is higher for the long window than for the short window at 0.25 Hz (*p* < 0.001) and 4 Hz (*p* = 0.001). For the long-window condition, the modulation sensitivity is constant up to 2 Hz (*p* > 0.153, *n. s*.) but declines with the higher temporal frequencies (*p* < 0.002). By comparison, the modulation sensitivity for the short-window condition is different between all corresponding pairs of the temporal frequencies (*p* < 0.006) except between 0.5 and 2 Hz (*p* = 0.445, *n. s*.), thereby indicating an inverse U-shaped function with a peak around 1 Hz.

#### Systems analysis

To quantitatively analyze changes in the velocity modulation sensitivity function between window sizes, we fitted the mean and individual data with a temporal MTF (Motoyoshi, 2011) obtained by Fourier transforming the impulse response function described by Equation (1). This kind of function has been employed in motion energy models (Adelson and Bergen, 1985; Watson, 1986). Specifically, in the present context, the impulse response describes the system's response over time to a very brief increment in the speed of a stimulus that was already present and moving at uniform speed.

(1) $$\begin{array}{r} f\left(t\right)={\left(kt\right)}^{n}exp\left(-kt\right)\left[1/n!-B{\left(kt\right)}^{2}/\left(n+2\right)!\right] \end{array}$$

Here, *n, k*, and *B* are free parameters that respectively determine tuning width in the frequency domain, filter center frequency, and the weighting of the negative phase relative to the first positive phase, or “transient factor.” As the transient factor increases, lower-band power attenuates in the frequency domain and the MTF curve shifts to a bandpass shape whereas the curve shifts to a lowpass shape as the transient factor decreases. We fitted the Fourier transform of this function to the data of each observer by means of the least-square method on the log scale with a scaling factor denoting the overall sensitivity (amplitude). The fitting was good for all observers. On average, the RMS error of the fitting was 0.05 on a log scale, and the correlation coefficient between the fitted and observed data was 0.97.

Figure 2B shows the average estimates of *n, k*, transient factor, and amplitude, each of which is 1.02, 12.76, 0.29, and 0.007 in the long window and 1.01, 8.07, 0.79, and 0.003 in the short-window condition respectively. A significant difference is found between the window conditions for *k* (*t*-test in log scale: *p* = 0.015) and transient factor (*t*-test in log scale: *p* = 0.045) but not for *n* (*t*-test in log scale: p = 0.526, *n. s*.) and amplitude (*t*-test in log scale: *p* = 0.546, *n. s*.). This indicates that the MTF curve has a lower central frequency and more bandpass shape in the short-window condition compared to the long-window condition, but tuning width and overall sensitivity are almost constant irrespective of the window condition. This tendency is observed across observers as illustrated in right panels of Figure 2A. Modulation sensitivity is constant or rises up slightly to 1–2 Hz (4 Hz only for SM), but sensitivity drops at higher temporal frequencies for the long window whereas it peaks at 1 Hz with a large reduction in the lower temporal frequency for the short window. Figure 2C shows the temporal impulse response function obtained by taking the inverse Fourier transform of the fitted MTF [note that we assume the impulse response function to be a biphasic form as applied by Equation (1) on the basis of a biphasic response of motion detectors (Glasser et al., 2011)]. The blue and red curves depict the system impulse response in the long- and short-window conditions respectively. The large negative phase appears following the positive phase in the short window (transient factor = 0.79) but becomes much smaller in the long-window condition (transient factor = 0.29). Such temporal characteristics correspond to lowpass and bandpass functional curves in the frequency domain respectively. Results indicate that, in line with results from previous studies (Snowden et al., 1991; Werkhoven et al., 1992), velocity modulation sensitivity for long-window grating stimuli is lowpass function with respect to the temporal frequency of the velocity modulation. However, velocity modulation sensitivity becomes bandpass with a peak around 1 Hz for comparatively short-window stimuli.

### Temporal-frequency modulation of luminance flicker

In principle, the bandpass function reported above could merely reflect a sensitivity to temporal-frequency modulation (FM) in the pixel luminance of the drifting grating rather than a sensitivity to velocity changes in the motion domain. To test the contribution of luminance FM, we measured detection thresholds for the temporal-frequency modulation of luminance flicker in a disk stimulus. We decided against using a grating stimulus because a phase-inverting (i.e., counterphasing) grating stimulus is mathematically equivalent to two gratings drifting in opposite directions. Depending on the course of the observer's voluntary attention, counterphasing gratings can elicit momentary percepts of directional motion along either of the two grating components (Cavanagh, 1992). We therefore elected to use a stimulus—a flickering disk—that does not elicit apparent motion to avoid confounding attentive motion tracking with sensitivity to luminance FM. Disk stimuli were displayed on each side of fixation for 2 s where each disk was repetitively inverted in luminance between brighter and darker with respect to the mean luminance background at a temporal frequency of 10.7 Hz. However, a sinusoidal temporal-frequency modulation from one of several temporal frequencies (0.25–8 Hz) was added to one of the two disks. Stimulus diameter was 2.1°—the same as a cycle of the grating stimuli used in the velocity modulation experiment—and the periphery was blurred by a cosine ramp over a spatial extent of 0.5°. Eccentricity (2.1°) and luminance contrast (0.4) matched those of the main velocity modulation experiment. Observers indicated which of the two disk stimuli contained a temporal-frequency modulation of luminance flicker, and detection thresholds were estimated after the measurements. All other experimental procedures were identical to those in the main experiment. Three of the original observers as well as another participant took part in these measurements.

**Figure 3:**
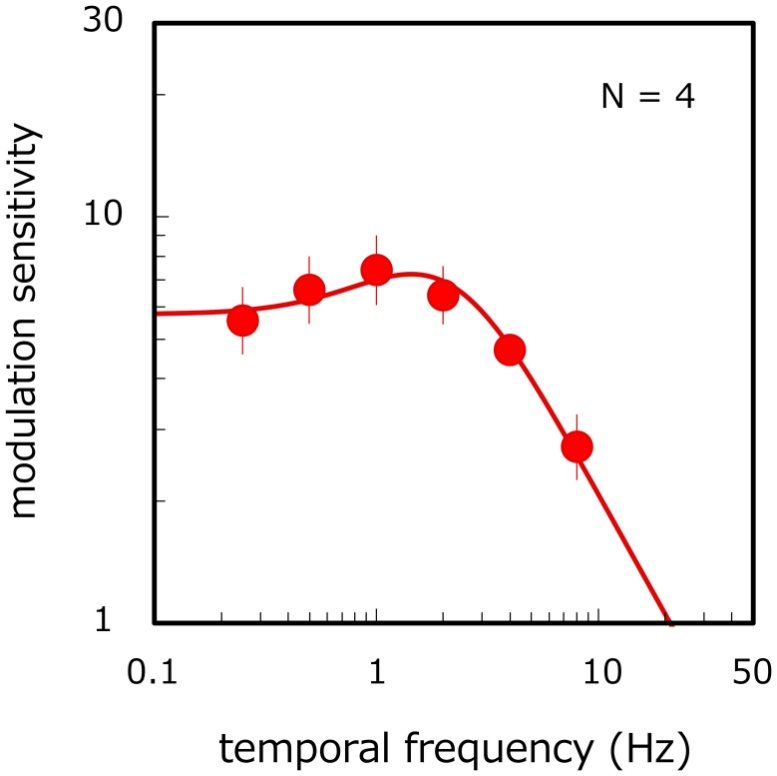
Temporal-frequency modulation sensitivity for luminance flicker. Filled circles and MTF curve show results averaged across observers. Error bars represent ±1 SE across observers.

Figure 3 shows modulation sensitivity for luminance flicker as a function of the modulation frequency. An ANOVA shows the main effect of modulation frequency [ANOVA: *F*_(5, 15)_ = 7.377, *p* = 0.001]. Modulation sensitivity is constant up to 2 Hz [*p* > 0.134, *n. s*.] but falls at higher temporal frequencies (*p* < 0.047), thereby indicating a lowpass shape function. We conducted a systems analysis again. On average, the RMS error of the fitting was 0.07, and the correlation coefficient between the fitted and observed data was 0.96. The average estimates of *n, k*, transient factor, and amplitude were 1.01, 15.02, 0.001, and 0.35 respectively. The value of the transient factor is not statistically different from its value for long-window stimuli in the main experiment (unpaired *t*-test in log scale: *p* = 0.856, n. s.) but is significantly lower statistically than its value for short-window stimuli (unpaired *t*-test in log scale: *p* = 0.035). While the shape of the data does not appear to be strictly lowpass, statistical analyses nonetheless show that a similar lowpass shape is observed for long-window stimuli in the main experiment. These results demonstrate that the bandpass function obtained in the main experiment depends on velocity modulation but not on the temporal-frequency modulation of luminance components.

## Experiment 2

The results of Experiment 1 imply that short windows reduce the information available about slow modulations. This suggests that some form of spatial integration is required for best performance with slow modulations. And one possible interpretation is that attentive tracking is required, and needs to be deployed over quite long distances. Hence in Experiment 2, we directly examined the effect of focal attention. To this end, detection thresholds were again measured for velocity modulation for long-window stimuli with and without a concurrent letter identification task (single/dual task). If attention plays an important role in detecting slow-rate velocity changes, then we expect that reducing observer attention with a dual task would cause the lowpass sensitivity function to become more bandpass.

### Methods

Velocity modulation sensitivity was measured in the same way as Experiment 1 but only for long-window stimuli. During the stimulus-presentation period of 2 s, a rapid-serial visual presentation (RSVP) display appeared concurrently in the center of the screen instead of the fixation point. We used the RSVP display in order to confine the observers' attentional resources throughout stimulus presentation. In the RSVP display, 12 capital alphabetical letters (excluding I, O, Q, Y, Z) were serially presented every 83 ms and separated by a blank interval of 83 ms (6.1 Hz). Each letter was drawn in Arial font in black and subtended 0.36 × 0.36°. Two letters were replaced by two numbers chosen at random between 1 and 9. One of the two numbers appeared somewhere within the 2nd-to-5th letter sequence, and the other number appeared within the 7th-to-11th letter sequence.

Single and dual task modes were tested in separate blocks. In single-task blocks, observers viewed the display with a steady fixation on the central RSVP letters and indicated the grating with a velocity modulation. Observers were instructed to concentrate on detecting velocity modulation while gazing at the central letters. In dual-task blocks, observers were first asked to identify the two numbers in the central RSVP display. If they identified both numbers by pressing buttons in the correct order, observers then indicated the grating with the modulated velocity. On average, observers were able to respond within a few seconds after the stimulus presentation. Auditory feedback was given on each task. Observers were instructed to keep letter identification performance as high as possible. In the dual-task mode, all observers received practice trials in advance and only trials in which observers correctly identified the central numbers were used for subsequent analysis. The average proportion correct of letter identification was 96.4%.

### Results

**Figure 4:**
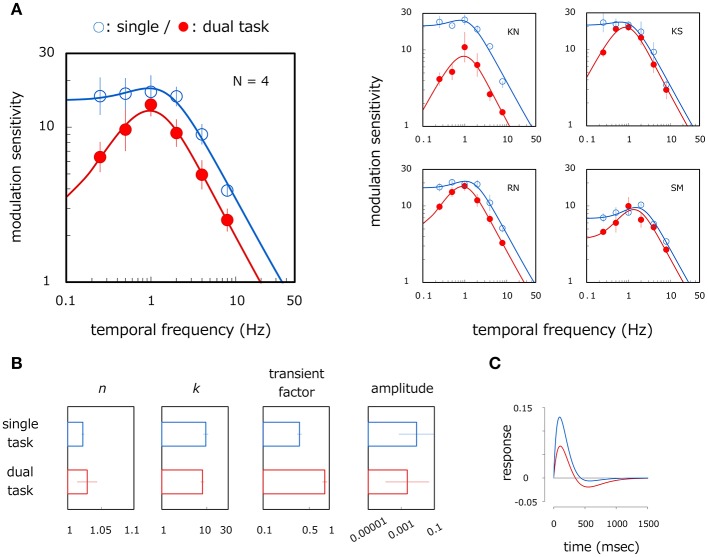
Results of Experiment 2. **(A)** Velocity modulation sensitivity for different task modes. Left panel shows results averaged across observers. Right panels show results for each observer. Open and filled circles show modulation sensitivity as a function of temporal frequency of velocity modulation for single- and dual-task modes respectively. Each curve represents the fitted MTF. Error bars represent ±1 SE across observers or 400 bootstrap samples within observer. **(B)** Estimated parameters of the MTF for single- and dual-task modes. Error bars represent ±1 SE across observers. **(C)** Blue and red curves show estimates of temporal impulse response function for the single- and dual-task modes respectively.

The left panel of Figure 4A shows average velocity modulation sensitivity as a function of modulation frequency. Open and filled circles show results for the single- and dual-task modes respectively. The smooth curve is the MTF fitted to the measured data. A two-way ANOVA reveals the main effect of modulation frequency [ANOVA: *F*_(5, 15)_ = 43.391, *p* < 0.001] and the interaction between modulation frequency and task mode [ANOVA: *F*_(5, 15)_ = 7.189, *p* = 0.001]. Modulation sensitivity is higher in the single-task mode than in the dual-task mode at 0.25 (*p* = 0.002), 0.5 (*p* = 0.045), 2 (*p* = 0.040), and 4 Hz (*p* = 0.025). In the single-task mode, modulation sensitivity is constant up to 2 Hz (*p* > 0.625, *n. s*.) but declines at higher temporal frequencies (*p* < 0.004). However, modulation sensitivity in the dual-task mode is different for all corresponding pairs of temporal frequencies (*p* < 0.015) with the exception of between 0.5 and 2 Hz [*p* = 0.704, *n. s*.] and between 0.25 and 4 Hz (*p* = 0.069: *n. s*.), thereby indicating a bandpass shape with a peak around 1 Hz. Although overall sensitivity is not significantly different between task modes [ANOVA: *F*_(1, 3)_ = 5.273, *p* = 0.105, *n. s*.], a reduction was observed over a wide range of temporal frequencies including higher bands in the dual-task mode.

#### Systems analysis

We analyzed the differences in velocity modulation sensitivity between task modes in the same way as Experiment 1. We fitted the MTF—the Fourier transform of the impulse response function (1)—to the data of each observer. Fitting was good for all observers. On average, the RMS error of the fitting was 0.04, and the correlation coefficient between the fitted and observed data was 0.98. Figure 4B shows average estimates of *n, k*, transient factor, and amplitude (1.02, 9.83, 0.36, and 0.009 in the single task, and 1.03, 8.20, 0.87, and 0.005 in the dual task). We found significant differences between task modes for the transient factor (*t*-test in log scale: *p* = 0.016) but not for *n* (*t*-test in log scale: *p* = 0.719, *n. s*.), *k* (*t*-test in log scale: *p* = 0.015), and amplitude (*t*-test in log scale: *p* = 0.190, *n. s*.). Results imply that the MTF curve becomes more bandpass in the dual-task mode in comparison to the single-task mode but that tuning width, filter center frequency, and overall sensitivity remain almost constant across task modes. This tendency remains roughly constant across observers as we illustrate in the right panels of Figure 4A. Modulation sensitivity is constant or rises slightly (typical for SM) up to 1–2 Hz but drops at higher temporal frequencies in the single-task mode. Sensitivity peaks at 1 Hz with a large reduction at lower temporal frequencies in the dual-task mode. The estimated curves of the temporal impulse response functions also show a large negative phase in the dual-task mode but only a very small negative phase in the single-task mode (Figure 4C). Together, results from Experiment 1 and 2 show that sensitivity is lowpass if grating windows are elongated in the direction of drift and if observers can attend exclusively to the stimuli, but sensitivity can readily become bandpass. These results show that the high sensitivity to the slow-rate velocity modulation is a product of attentive tracking to the moving stimuli.

## Experiment 3

In the last experiment, we tried to provide another line of evidence that attention mediates detection of slow-rate velocity modulation. As it is difficult to track multiple moving stimuli simultaneously (Pylyshyn and Storm, 1988; Yantis, 1992), visual search tasks in such displays would reduce the effects of attentive tracking and reveal the contribution of low-level mechanisms as a whole. We measured detectability for a velocity-modulated target embedded in a set of constant-velocity distracters. If a particular rate of velocity modulation is detected efficiently by low-level mechanisms, we would expect sensitivity for detecting velocity-modulated targets to be a bandpass function of modulation frequency.

### Methods

**Figure 5:**
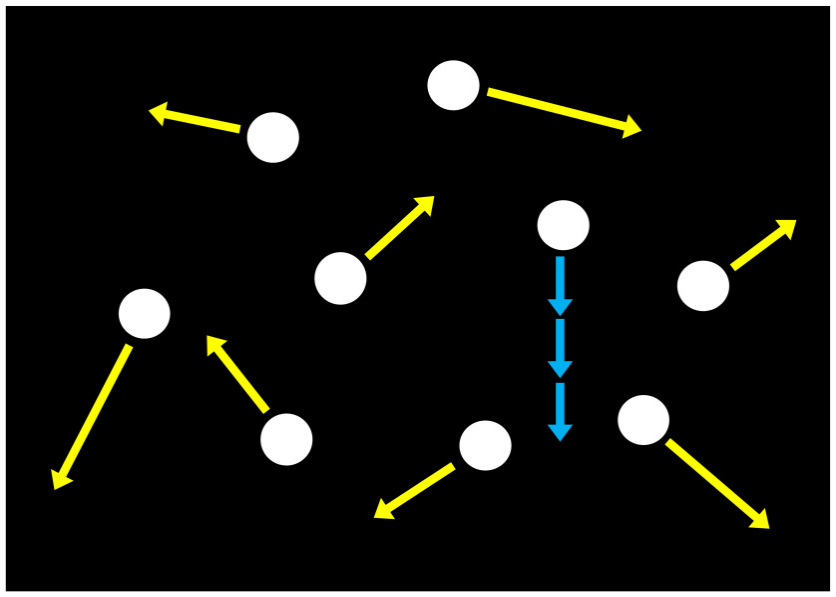
Schematic of the search display used in Experiment 3. Nine luminance dots moved at different velocities and did not to overlap with each other as denoted by yellow arrows. In target-present trials, an average-speeded dot is given a sinusoidal velocity modulation at one of various temporal frequencies (0.25–8 Hz) as shown by blue arrows.

The search display was composed of nine luminance dots (0.43° in diameter, 100.1 cd/m^2^), each of which was located at a random position on the dark background (17.0 × 17.0°) and moved at different velocities where speeds were distributed uniformly from 6.8 to 10.2 degree/s and directions were randomly determined (Figure 5). Dots did not to overlap with each other and trajectories wrapped around the display—that is, dots disappeared when they reached the edge of the background, reappeared in the corresponding diagonal position, and resumed their original trajectory on a parallel path. In half the trials, a sinusoidal velocity modulation at one of various temporal frequencies (0.25–8 Hz) was added to a dot moving at 8.5 degree/s (target-present trials). The velocity modulation depth was fixed at 0.6 and the temporal phase of the velocity modulation was randomized. The various modulation temporal frequencies were counterbalanced in each experimental block. In the other half of the trials, all the dots moved at different but constant velocities (target-absent trials).

The experiment was conducted in a dark room. In each trial, dot stimuli were presented for 2 s. Observers viewed them freely and, after the stimulus presentation, indicated the presence/absence of the velocity-modulated target by pressing a key. Auditory feedback was given on correct and incorrect responses.

### Results

**Figure 6:**
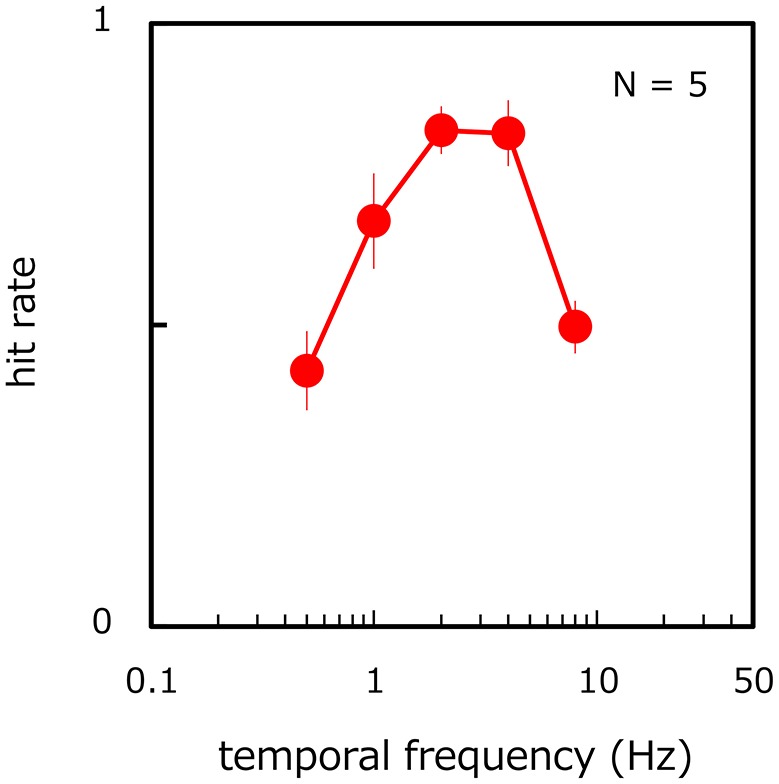
Hit rate of target presence/absence as a function of the temporal frequency of target velocity modulation. Error bars represent ±1 SE across observers.

Figure 6 shows the proportion of correct trials in target-present trials as a function of the temporal frequency of target velocity modulation. The proportion of incorrect trials in target-absent trials was 11.6% averaged across observers (SE = 2.32%). On the hit rate, ANOVA reveals the main effect of the modulation frequency [ANOVA: *F*_(4, 16)_ = 18.476, *p* < 0.001]. The hit rate was different between all corresponding pairs of temporal frequencies (*p* < 0.027) except between 2 and 4 Hz (*p* > 0.935, *n. s*.) and between 0.5 and 8 Hz (*p* > 0.245, *n. s*.), thereby revealing a bandpass function with a peak frequency of around 2 Hz. For all observers, the hit-rate function had a peak at 2 or 4 Hz but all performances were impaired at lower and higher temporal frequencies. These results are consistent with the notion that the visual system processes relatively fast-rate velocity modulations most efficiently when it is difficult to continually track each of the moving stimuli by focal attention.

## General discussions

The present study provides a quantitative description of detection sensitivity for velocity modulation in moving stimuli. Modulation sensitivity was found to be a lowpass function of modulation temporal frequency provided that, as indicated by past studies (Snowden et al., 1991; Werkhoven et al., 1992), observers could easily track moving stimuli with attention. However, we found that sensitivity function becomes bandpass (peak at ~1 Hz) if attentive tracking is made difficult by experimentally manipulating stimulus size (Experiment 1) or attentional resources (Experiment 2). Bandpass sensitivity appeared to be independent of the modulation temporal frequency of luminance components (Experiment 1). An additional visual search experiment revealed that relatively fast-rate velocity modulation (~2–4 Hz) was detected most easily among constant motions (Experiment 3), thereby suggesting that rapid accelerations are processed highly efficiently despite little or no contribution from attentive tracking. These results can be interpreted as evidence that the visual system is directly sensitive to accelerations/decelerations and processes changes in velocity at the preattentive level.

The present results show that high sensitivity for sluggish accelerations is likely the result of attentive tracking—a finding consistent with past studies that have reported lowpass sensitivity for acceleration in single moving objects like dots (Werkhoven et al., 1992). Watamaniuk and Heinen (2003) argued that smooth pursuit of accelerating stimuli depends on stimulus velocity but also depends on acceleration within a certain range of comparatively low accelerations. Mueller et al. (2016) directly showed that acceleration sensitivity improves for moving stimuli that are easily tracked by attention or by smooth pursuit. Such results suggest that attentive tracking is the primary factor contributing to the detection of sluggish accelerations. Acceleration detection has been attributed to cognitive processes that compare velocities over time (Gottsdanker et al., 1961; Schmerler, 1976; Brouwer et al., 2002), and this cognitive framework can account for the effects of attentive tracking. That is, attentive tracking enables observers to determine instantaneous velocity at different moments in time and to compare these velocities in working memory.

The present results might indicate further possibility that peripheral and central attentions facilitated slow and fast motion processing, respectively, and contributed to a lowpass and bandpass function of the modulation sensitivity independently from the role of attentive tracking. This idea is consistent with the long- and short-window conditions in Experiment 1 and the single and dual tasks in Experiment 2 given that the central RSVP task withdraws attention from the periphery. The bandpass sensitivity obtained in Experiment 3 seems inconsistent with the fact that stimuli moved over a relatively wide range, but observers freely moved their gaze in Experiment 3 and engaged in focal rather than broad attention to track moving stimuli. Therefore, an attention mode that functions differently in peripheral and central vision could possible determine the shape of the MTF for velocity modulation.

As mentioned in the Introduction, preattentive acceleration detection can be conceptualized in terms of a biphasic—or bandpass—temporal response applied to otherwise well-known motion detectors. The biphasic response may reflect a mixture of non-linear processes, including adaptation, but a large part of our data seem consistent with the assumption that a biphasic response imposed on the output of motion detectors effectively acts as a temporal filter capable of detecting acceleration and deceleration. In favor of this view, it has been reported that motion-induced shift of position (Roach and McGraw, 2009; Cavanagh and Anstis, 2013) or timing (Nagai et al., 2000; Öǧmen et al., 2004) is strengthened by acceleration and even reversed by deceleration. For example, Roach and McGraw (2009) showed that flash drag effects overshoot immediately after motion onset whereas they undershoot after motion offset. These findings suggest that acceleration signals play a significant role not only in motion perception but also in the spatiotemporal localization of moving stimuli.

Experiments in the present study have shown that the detection of velocity modulation in moving stimuli is a bandpass function of the modulation's temporal frequency but that detection peaks at different frequencies for different experiments: ~1 Hz for detection thresholds with drift gratings (Experiments 1 and 2) and ~2–4 Hz for target detection rate in visual search (Experiment 3). Differences between peak detection frequencies may depend on differences in stimulus type and/or on the nature of the behavioral task in each experiment. Another possibility, however, is that, in cases where pairs of moving stimuli were displayed simultaneously, observers were able to directly compare velocities and use this velocity differential as a cue to detect sluggish accelerations. For example, perhaps observers could infer slow-velocity modulation by comparing the maximum velocity of the modulated grating to the constant velocity of the unmodulated grating. Such a strategy could have artificially boosted sensitivity to low modulation temporal frequencies. By contrast, such cues were not available in the search display that contained multiple stimuli with various velocities. In line with this hypothesis, bandpass sensitivity in the search experiment was found to be sharper and shifted to higher frequencies relative to sensitivity in the two-grating experiments. This sharper and higher-frequency bandpass sensitivity may reflect the underlying activity of a rapid biphasic unit that operates directly on relatively fast accelerations.

## Ethics statement

This study was carried out in accordance with the recommendations of guidelines on research ethics by The Japanese Psychonomic Society with written informed consent from all subjects. All subjects gave written informed consent in accordance with the Declaration of Helsinki. The protocol was approved by the ethics committee of the University of Tokyo.

## Author contributions

RN and IM designed the study. RN performed experiments, analyzed data. RN and IM drafted the manuscript.

### Conflict of interest statement

The authors declare that the research was conducted in the absence of any commercial or financial relationships that could be construed as a potential conflict of interest.
